# A deep learning framework for real‐time 3D model registration in robot‐assisted laparoscopic surgery

**DOI:** 10.1002/rcs.2387

**Published:** 2022-03-13

**Authors:** Erica Padovan, Giorgia Marullo, Leonardo Tanzi, Pietro Piazzolla, Sandro Moos, Francesco Porpiglia, Enrico Vezzetti

**Affiliations:** ^1^ Department of Management, Production and Design Engineering Polytechnic University of Turin Turin Italy; ^2^ Department of Oncology, Division of Urology, School of Medicine University of Turin Turin Italy

**Keywords:** abdominal, Kidney, prostate

## Abstract

**Introduction:**

The current study presents a deep learning framework to determine, in real‐time, position and rotation of a target organ from an endoscopic video. These inferred data are used to overlay the 3D model of patient's organ over its real counterpart. The resulting augmented video flow is streamed back to the surgeon as a support during laparoscopic robot‐assisted procedures.

**Methods:**

This framework exploits semantic segmentation and, thereafter, two techniques, based on Convolutional Neural Networks and motion analysis, were used to infer the rotation.

**Results:**

The segmentation shows optimal accuracies, with a mean IoU score greater than 80% in all tests. Different performance levels are obtained for rotation, depending on the surgical procedure.

**Discussion:**

Even if the presented methodology has various degrees of precision depending on the testing scenario, this work sets the first step for the adoption of deep learning and augmented reality to generalise the automatic registration process.

## 1 INTRODUCTION

In recent years, artificial intelligence (AI) and deep learning (DL) are becoming increasingly common due to the widespread digitalisation that has occurred in many fields. This spreading resulted in collecting a huge amount of data enabling their better accessibility and their exploitation for automated processes, especially pertaining to AI, in a general perspective of efficiency improvement. Since medicine and health care have always been among the areas that benefit the most from progress, several efforts are still being made to implement the AI technology for practical medical treatments and health care management.^1^ In particular, the development of F (CNNs) has allowed the machines to learn useful tasks from images, such as detecting and characterising suspicious patterns from radiographs or provide diagnosis and prognosis in different medical fields.^2^ Urology was one of the first adopters of artificial intelligence for object detection, image classification, image segmentation, skills assessment, and outcome prediction for complex urologic procedures.^2^ The advantages of DL have been exploited also in laparoscopic surgery to support the surgeon during certain procedures. Laparoscopic surgery is currently one of the most performed surgical technique as it is minimally invasive yet, despite its benefits, it leads to a lack of direct tactile and visual perception of the surgical site.^3^ Furthermore, the surgical scene is typically complex and dynamic, resulting in events that contribute to occluding, blurring, and defacing the view.^4^ These drawbacks can be mitigated exploiting augmented reality (AR), which allows real‐time overlapping between computer‐generated images or 3D models, comprising of anatomical structures of interest, and the real environment. The main challenge of an AR application is the registration process, namely, the accurate alignment of the virtual model with its physical counterpart, in terms of spatial and rotational coordinates.^5^ The registration phase is fundamental to accurately discern the structures of interest from noise and background and ensure that the augmented view conforms as closely as possible to the real surgical scene.

In order to superimpose the preoperative model on the intraoperative image, it is necessary to know the six degrees of freedom, that is, three parameters for position and three for rotation along the three *X*, *Y*, and *Z* axes. These six parameters allow to univocally determine position and orientation of an object in three‐dimensional space and are referred to as its 6D pose. As higher demand for new scenarios has risen, this topic has been highly investigated in recent years. Different kind of information is used to address this task, from texture, colour, and geometry to depth information, since lately a great development of hardware has occurred, nevertheless depth‐based approaches have not been considered for this study. After a comprehensive literature review of the current methodologies for 6D pose estimation, the top performers were selected. Considering these approaches, a novel framework was proposed to support the surgeon in the visualization and localization of surgical targets and critical structures of interest, such as endophytic or posteriorly located tumours, that would otherwise be arduously identified. To this purpose, a virtual 3D model of the patient's organ, obtained from the preoperative imaging,^6^ was intraoperatively and automatically superimposed on the endoscopic view and displayed on the screen of the surgery robotic system in use, to guide the surgeon during the incision. Given the variety of solutions presented in the literature using different data and different devices, this methodology is an easy‐to‐integrate alternative, whatever robotic surgical system is in use, as it does not add hardware complexity.

The core of the framework is to recover the 6D position of the organ in real‐time, relying solely on endoscopic video RGB frames. In other words, to address this task, we could rely only on the elements present in the endoscopic video, that is the rigid instruments, which are inserted and remain static during the operation, and the organs, which are deformable and not easily distinguishable from the surrounding tissues.

Three phases could be defined: (1) localization, (2) registration, and (3) tracking.

To localise the different elements in the scene, a first step consisted of semantic segmentation (SegmentationCNN), consisting of assigning class labels to the pixels of the image, obtaining colour‐coded segmentation masks of the classified objects, while, in a second phase, a CNN addressed the rotation estimation (RotationCNN) to perform the registration. Depending on the element used as reference, the RotationCNN showed a lack of robustness. When rigid instruments figured out, it proved to be a stand‐alone solution, and it was possible to indirectly retrieve the 6D organ position using some specific surgical instruments as references. When it was tested on organs, it was necessary to add to the methodology an alternative third approach, based on Optical Flow (OF), to mitigate the lack of robustness of the RotationCNN. To this aim, since consecutive frames have high correlation, instead of inferring the 6D pose for each frame through the RotationCNN, the Optical Flow, starting from an initial frame, deduces the 6D pose by performing a pixel wise tracking of the organ. In this way the temporal information of the video flow is exploited. The SegmentationCNN was tested by evaluating the Intersection‐over‐Union (IoU) metric, while the RotationCNN and the OF, were tested by comparing the predicted values with the actual ones. The presented framework was conceived as a generalized procedure capable of handling different surgical operations. To demonstrate the generalisation capability in terms of both the surgical procedure and the element used for the registration, two surgical operations, namely the Robot‐Assisted Radical Prostatectomy (RARP) and the Robot‐Assisted Partial Nephrectomy (RAPN), were considered, and three case studies were taken into consideration: the first involving a static artificial element, such as the catheter, while the second and the third relying solely on organs, that is, the prostate and the kidney.

The paper is organised as follows: Section 2 conveys an extensive overview on recent 6D pose estimation systems; Section 3 gives a description of this generalized framework, subdivided into: datasets presentation (Section 3.1), location estimation (Section 3.2), and rotation inferring (Section 3.3), training process and testing metrics (Section 3.4), Case Studies in analysis (Section 3.5); Section 4 discusses the results obtained; Section 5 discusses the study; finally, Section 6 gives a brief conclusion of the work.

## 2 RELATED WORKS

6D position estimation is an essential task in many Computer Vision (CV) applications. It concerns, as well as the surgical field,^7^, ^8^ robotics,^9^ autonomous driving,^10^ and virtual/augmented reality (VR/AR) applications^11^ and is extensively used in the entertainment industry.^10^ The problem itself is simple and consists of determining the 3D rotation and translation of an object which shape is known in relation to the camera, using details observable from the reference 2D image. However, achieving a solution to this problem is not trivial.^9^ Firstly, due to auto‐occlusions or symmetries, the objects cannot be clearly and unequivocally identifiable. Moreover, the image conditions are not always optimal in term of lighting and occlusions between the objects represented in the picture.^12^, ^13^ In these situations, it is often necessary to add an earlier stage of object detection or localization to distinguish the area of the image which contains the object, before estimating its position.

Although this problem has been studied for many years, the advent of DL gave a renewed push to find a solution, as well as it fostered the research in other fields of application, such as the medical^14^, ^15^, ^16^ or face recognition^17^, ^18^ domains. Old pose estimation methods were based on geometrical approaches, as for example, Feature‐based methods,^19^ which tried to establish correspondences between 3D models and 2D images of objects by using manually annotated local features. With texture‐less or geometrically complex objects, it was not easy to select local features. In these cases, even though the matching phase usually took much time, it might fails and provides a not always accurate result.^20^

In opposition to these methods, researchers introduced Template‐based methods,^21^, ^22^ which represented the 2D object from different points of view and compared these representations with the original image to establish the position and orientation. These approaches were very susceptible to variations in lighting and occlusions even if they could manage texture‐less objects and required many comparisons to reach a certain accuracy level, increasing the execution time.^11^ With the diffusion of DL, researchers improved traditional methods by introducing Learning‐based methods, making them more efficient and performing. The basic idea of these systems involves CNNs to learn a mapping function between images with three‐dimensional position annotations, and object 6D position. Some of these systems^23^, ^24^ employ a CNN to predict the 3D bounding box, and then a PnP algorithm, which calculate the 6D position from matches between 2D features on the test image and 3D points on the CAD model.^25^ Other types of Learning‐based methods, instead, need only a CNN to resolve a classification^26^, ^27^ or a regression problem.^28^, ^29^ For this reason, Learning‐based methods are referred as Bounding box prediction and PnP algorithm‐based, Classification‐based, and Regression‐based methods, respectively. These methods can reach very high levels of precision, but they need many data to train the network accurately and to be able to work well in real cases. Alternatively, CNNs can be used to execute the most critical steps of traditional methods to join the advantages of the various strategies into the final solution.^20^

After identifying the golden standards of 6D pose estimation in various fields, works related to the laparoscopic surgery augmentation specific purpose were investigated. This second phase of the review aimed to analyse the surgical goal to understand the variety of contexts of use and to find out if any effective method was already proposed.

The most popular 6D pose estimation algorithms, mainly focus on objects with distinctive shapes or textures. These characteristics make the process less complex than the one here addressed. As a matter of fact, anatomical structures do not have easily recognisable patterns, yet their texture and shape depend on many factors, from the anatomy of the patient to even his/her position or pathology. For this reason, every approach used for anatomical purposes should be produced as intended to be generalisable for different patients and, at the same time, sufficiently accurate for the specific case. Most of the medical applications are referred to abdomen laparoscopic procedures; since the real‐time video of the surgical scene is captured and displayed on an external monitor, it can be easily accessed to process the intraoperative information.

Applications were proposed for kidneys, prostate, uterus, and liver.^4^ Depending on the organ some approaches were more suitable than others.^30^, ^31^ The aim of these applications is to perform a first registration of the virtual 3D model on the endoscopic view and the tracking phase, in which the model adapts its position and orientation in accordance with the real organ.

These tasks are challenging mainly because of the lack of stationery features to fall back on, as there are discrepancies between the preoperatively obtained data and the intraoperative real‐time video flow. Performing the pneumoperitoneum, namely the insufflation of air or another gas in the peritoneal cavity, the abdominal cavity undergoes some deformations that make the target in the scene different from the one from the preoperative images. In addition to this, further deformations due to tool interactions, as well as smoke and blood presence, must be dealt with. For this reason, keeping track of the target position and recovering it if needed, is fundamental.

The first and easiest method of anchoring is the manual registration.^32^, ^33^ Efforts have been made to minimise the human contribute, by introducing semi‐automatic systems, pause and editing approaches,^32^, ^34^ deformation handling^30^, ^33^ and fully automatic algorithms.^8^, ^35^

For what concerns the tracking phase, the artificial model has to follow the organ movements in real‐time. This task can be performed following four main approaches^4^ which rely on techniques of increasing complexity. The first method, known as interactive, mainly exploits the human assistance, such as the pause and editing approach, analogously to the manual registration.^36^ In some cases, artificial or anatomical cues can be used as fiducial markers, to perform a point‐based match between the endoscopic organ and the virtual model, since they are visible both preoperatively and intraoperatively.^8^, ^33^ On the contrary, surface‐based methods focus on the intraoperative perspective rather than on preoperative data, because the surface is intraoperatively reconstructed directly on laparoscopic images and registered only at a later stage.^37^, ^38^ Finally, the volume‐based methodologies are the most complex ones, as they require an intraoperative imaging system in addition to the endoscope, to better locate the hidden structures.^39^ On the other hand, a double registration is needed, between preoperative and intraoperative volumes and between the preoperative volume and the endoscopic image.

Moreover, the previous matching techniques are mainly based on the rigidity hypothesis, meaning that organ deformations are not considered, hence the registration accuracy may be affected. To mitigate this problem, deformation models have been introduced to intraoperatively modify the preoperative model, aiming to fulfil the compromise of adaptability and accuracy.^30^, ^33^

Up to now, the most investigated methods in the surgical field require, in addition to the two‐dimensional RGB image, other types of data or other processing steps in the intraoperative stage. Although it is helpful to have supplemental data, the introduction of new sensors and possibly additional cameras is a cumbersome procedure that would lengthen the operating time of the procedure, as well as increase its cost, and introduces complications such as synchrony of these elements. This work intends to propose a more immediate and time‐saving novel methodology, able to perform a real‐time registration relying on RGB intraoperative monocular images only.

## 3 MATERIALS AND METHODS

**FIGURE 1:**
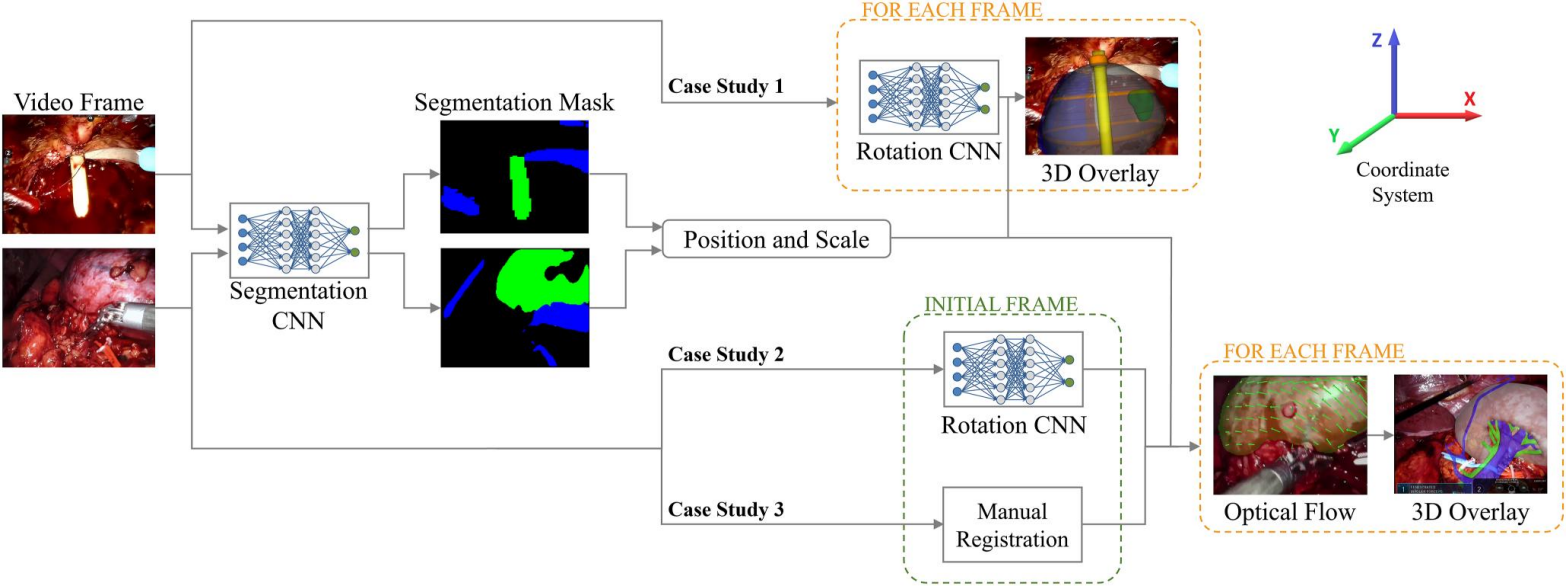
Schematic illustration of the system workflow. The video frame is fed to a Segmentation Convolutional Neural Networks (CNN) and a Rotation CNN, depending on the Case Study. Target's position and scale are obtained through the Segmentation CNN for every Case Study. Target's orientation is retrieved in different ways depending on the Case Study: Rotation CNN output the predicted rotation for each frame (Case Study 1); RotationCNN for registration in an initial frame and OF tracking for next frames (Case Study 2); Manual Registration in an initial frame and OF tracking for next frames (Case Study 3)

The methodology of the proposed framework, represented in Figure 1, consists of two subsequent phases and an alternative third phase. Firstly, the RGB image, captured by the endoscope, is fed to a first CNN to perform the semantic segmentation of the actors. A segmentation mask is returned, which distinguishes the objects in the scene, in this way the scale and position values of the element used as reference (coloured in green) are obtained. Contextually, the RGB image is fed to another CNN which returns the rotation values. Depending on whether the main actor is a rigid instrument or an organ, the RotationCNN could show different performances. For this reason, in some cases, it would be more accurate to deem this kind of registration as an initialisation step, performed on images in which the organ is fully exposed and well framed by the camera.

Thus, three different use cases were considered:

(1) When rigid instruments are present, such as the catheter (Case Study 1), the RotationCNN can be applied recursively, merging registration and tracking tasks in the same method. The reason why the rotation can be more easily retrieved, is that, among the three axes, only one is effectively unknown. Indeed, the rotation on the *Z*‐axis is irrelevant because of the catheter's symmetry, while the rotation along the *Y*‐axis can be successfully retrieved from the semantic map,^16^ as proven by results in Table 3. Thus, only the rotation along the *X*‐axis should be predicted.
(2, 3) When the organ is the only information to fall back on, different problems arise, such as organs' deformability, difficulty in distinguishing organ's texture from the surrounding tissues, and the fact that their rotation is bound to a fixed anatomical constraint. In this case, the RotationCNN should predict three rotations, as the one along the *Z*‐axis is now relevant and the one along the *Y*‐axis is not retrievable with precision from the semantic map. For this reason, when the organ has a particularly recognisable shape in ideal frames, such as the prostate (Case Study 2), the RotationCNN can be used for registration, while in other cases, when the organ presents a more complex structure, such as the kidney (Case Study 3), the initialisation should be manually performed. After the semi‐automated registration, the organ is tracked using a CV based approach, which exploits an OF motion analysis algorithm. This technique performs the pixel‐wise tracking of the organ to infer the frame‐to‐frame rotation and maintain the real‐time superimposition of the model correctly oriented.

The information from the two CNNs is combined for locating and orienting the 3D virtual model, to overlay it on the endoscopic view and displayed on the monitor of the robotic surgical system. The CNNs, used for these different tasks, are detailed in the next sub‐sections.

### 3.1 Datasets

The dataset creation strategy was different depending on the semantic segmentation or the rotation estimation CNNs. Both the approaches are detailed below.

#### 3.1.1 SegmentationCNN

The images employed for the dataset creation were acquired from nine endoscopic videos of different patients, recording the laparoscopic procedures in analysis. In particular, five videos referred to the prostate and, depending on the phase of the procedure, frames were extracted for Case Study 1 and 2, while 4 videos, referring to kidney, were used for Case Study 3. The average length of the procedures' videos was about 20 min, from which the most significant portions, from few seconds to 5 min each, were selected. Among the extracted frames, only those belonging to portions of the procedures in which the target object was well framed by the endoscope were actually used for datasets creation. The SegmentationCNN output, for each frame, a segmentation mask, containing three different classes, each marked with a unique ID.

#### 3.1.2 RotationCNN

To infer the organ rotations along the three axes through a Learning‐based approach, the CNN must be trained on images in which the organ rotation values are provided. This is a troublesome task to perform through manual rotational tagging on real images because it would be heavily operator‐dependent, hence unreliable. To avoid this source of uncertainty, a synthetic dataset was created in *Blender*, starting from the preoperative 3D virtual model of the target. The actual dataset creation is performed by a Python code, that renders synthetic images after computing scene changes such as: tools position and rotation, texture rotation, light changes, and organ rotation. The main advantage of the synthetic dataset is that the rotation of the organ is known, as it is controlled by the script itself.

### 3.2 Location and scale inferring

Given an RGB image, the first step of the organ *localization* is the semantic segmentation task, which aims to produce a matrix having the same size of the input image containing the predicted class identifiers corresponding to all pixels. To choose the network model, different attempts were made by combining pre‐trained base models and segmentation architectures. Among all the combinations between architecture and methods, UNet architecture with ResNet as base model has shown the best test accuracy, as usually happens for medical images.^40^

By applying the segmentation model to an intraoperative video, the target semantic mask can be visualised. To extract the target location, the irregular mask is circumscribed by a fitting ellipse, the centre of which is retrieved. Its coordinate is assigned to the centre of mass of the target's 3D model to perform the anchoring and the real‐time superimposition translation in the frame space. The same ellipse is used to infer the scale factor of the frame, meaning the distance from the camera, as its dimension is proportional to the one of the organ that is fitting.^16^

### 3.3 Rotation inferring

**FIGURE 2:**
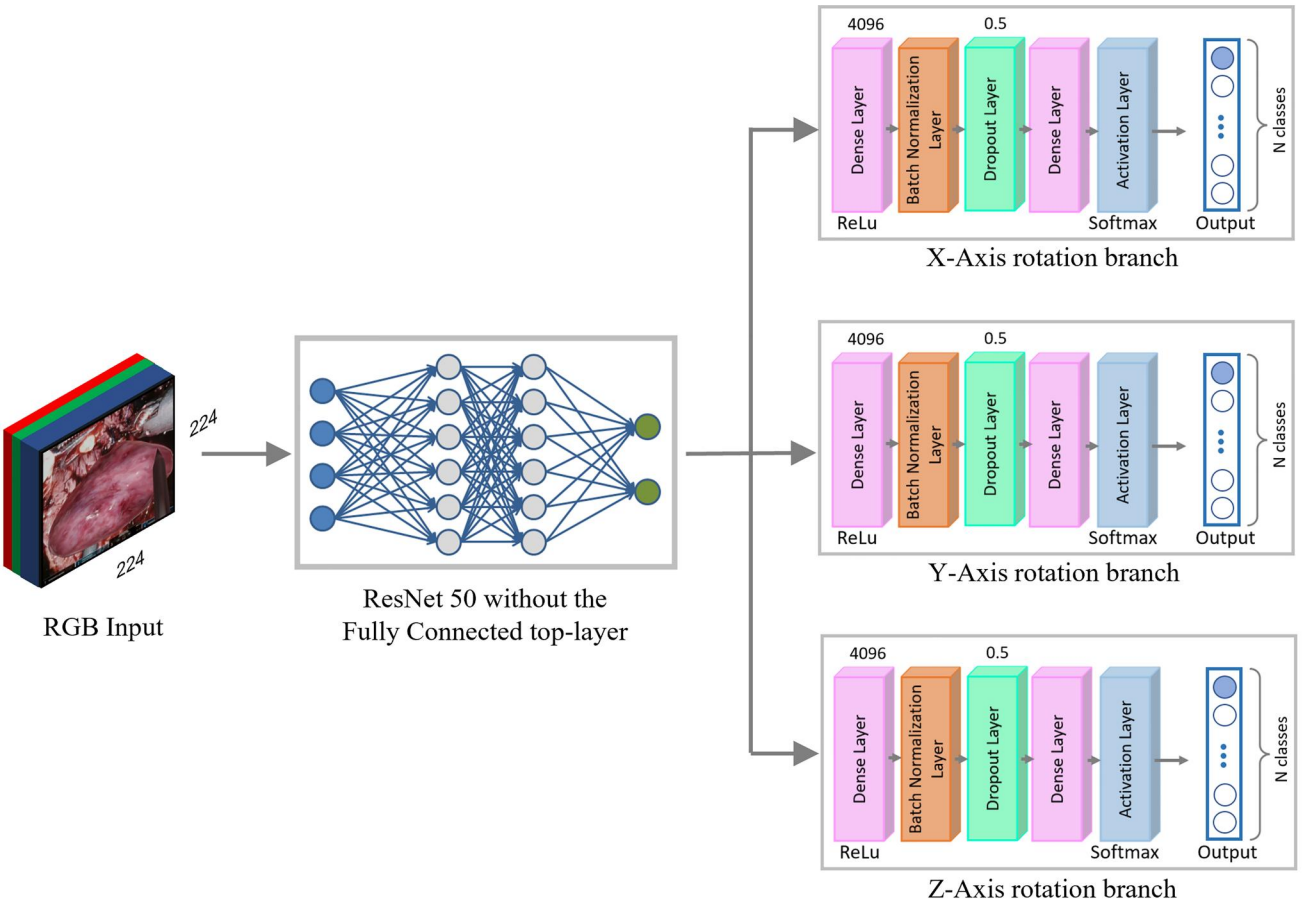
Rotation Neural Network overall architecture, which involves a modified version of the ResNet50 model, with a branch for each axis rotation

To perform the *registration* task, the rotation values must be first estimated. The RGB image, used as input of the SegmentationCNN, is fed to the RotationCNN, based on a modified version of ResNet50 model, with a different branch for each axis rotation. Every branch has the same structure, which contains: a *Dense Layer*, with 4096 neurons and a ReLU activation function; a *Batch Normalisation Layer*; a *Dropout Layer*; a *Classification Dense Layer*, with several neurons equal in number to the current axis classes, and an *Activation Layer*, which applies the *Softmax* activation function to the output. The estimation of the rotation along the three axes was solved as a classification problem. The set of possible rotation values along an axis were discretised in N classes, according to the range of orientation angles the target can assume during the surgical operation. The output neuron, corresponding to the highest probability value according to the *Softmax* activation function, fires and produces the corresponding rotation value. An overall representation of the Rotation Neural Network for the three axes rotation prediction case is presented in Figure 2.

#### 3.3.1 Optical flow

In case the target is the organ, the RotationCNN prediction is often not robust enough, hence must be manually corrected by the operator. The subsequent organ *tracking* is then addressed by the OF algorithm. The advantage of this method is the use of temporal information. As a matter of fact, the registration of the model, solved as a tracking task, is more easily retrievable, but not necessarily more accurate than if addressed by inferring the 6D pose within a single frame, due to the high correlation consecutive frames have. Through OF algorithm, motion analysis can be performed, by extracting the displacements, between consecutive frames, only regarding the organ pixels. OF returns two vectors, the magnitude and the angle direction of the displacement, used to display the displacement arrows on the image. The different distribution of the arrows direction on the organ contains the information about the rotation trajectory of the organ. Since the framework is applied when the organ is in situ, it is constrained to the surrounding anatomical structures. Therefore, the organ cannot move freely in the abdominal cavity, so the flow vectors do not describe pure translation, but they also contain rotation information. From this assumption, it was hypothesised that the magnitude of the vectors is an indicator of the rotation angle, while their direction is an indicator of the rotation axis. Once peculiar characteristics within the specific case of the organ movement were identified, according to the considered case studies (Section 3.5), it was observed that, when the displacement follows a particular pattern, it is possible to assume the rotation direction. Since these assumptions cannot be generalized, a heuristic was written to identify and quantify the organ rotation.

### 3.4 Training and metrics

The networks described in Sections 3.2 and 3.3 were trained by tailoring hyper‐parameters according to the available data of each case study (Section 3.5). To evaluate the CNNs, two different metrics have been involved.

The SegmentationCNN model was assessed by the *Intersection‐over‐Union* (IoU) metric, a diffuse metric for semantic segmentation, defined as:

$$\text{IoU}=\;\frac{\text{Overlap}\;\text{Area}}{\text{Union}\;\text{Area}}$$

where Overlap Area represents the intersection between the pixels belonging to the predicted mask and those belonging to the ground truth one, while Union Area represents the union between the pixels belonging to the predicted mask and those belonging to the ground truth one. The IoU score ranges from 0 to 1, where the edge values mean completely wrong and perfectly correct predictions, respectively.

The RotationCNN was evaluated by comparing the predicted and the actual rotation values on the *X*, *Y* and *Z* axes.

Both the CNNs ran on an NVIDIA Quadro P4000 GPU, involving the open‐source Keras library for neural networks, which is written in Python and run on top of TensorFlow.

### 3.5 Case studies

The proposed framework is designed to support the surgeon during critical phases of robot‐assisted laparoscopic surgical procedures. In particular, the urological field was considered, as it deals with organs with symmetrical, rounded and not very distinctive shapes and textures, that make them hardly distinguishable from the surrounding anatomical parts. These drawbacks can be mitigated by AR solutions. The opportunity to visualise 3D reconstructions overlayed on their real counterpart introduces several benefits as stated in literature,^4^, ^5^ as for example,: exploiting AR during preoperative planning for tailoring incisions and cutting plans; reducing surgery time; augmenting the surgeon's spatial perception of the surgical field, compromised by the use of the robot console, avoiding unnecessary manipulations or accidental injuries to inner organs; reducing the surgeon's distractions by allowing him or her to access the augmented information contextually without having to look for it in a different monitor.

In this work, the datasets are parts of intraoperative endoscopic videos from different patients and were provided by the "Division of Urology, University of Turin, San Luigi Gonzaga Hospital, Orbassano (Turin), Italy". AR has been used to support the surgeon during the following case studies.

#### 3.5.1 Robot‐Assisted Radical Prostatectomy (RARP)

Radical Prostatectomy removes the prostate gland and tissues surrounding it in case of prostate cancer. The precision of this procedure is fundamental for patient well‐being to avoid short and long‐term complications. The RARP procedure can be subdivided in five stages: (1) defatting and incision of the endopelvic fascia, (2) management of the bladder neck, (3) vase clamping and nerve‐sparing, (4) surgery by the prostatic apex, and (5) targeted biopsy. According to surgeons, phases 3 and 5 could benefit the most from AR, for which appropriate registration techniques have already been implemented.^3^ In both phases the overlay of the organ would be a significant help for the operating surgeon^41^: in the vase clamping and nerve‐sparing phase, AR is helpful for accurate nerves and vases localization, crucial for preserving patient's well‐being; while AR guided tumour localization in targeted biopsy is essential, in case of extra capsular tumour, to verify that the extra parenchyma part, which may have been in contact with the surrounding tissue, has not compromised it leading to metastases development later on. In phase 5, a catheter is inserted, and it could be used as a target to overlay the 3D model (Case Study 1). On the contrary, in phase 3 the 6D information have to be retrieved from the prostate (Case Study 2).

#### 3.5.2 Robot‐Assisted Partial Nephrectomy (RAPN)

In Partial Nephrectomy, only the affected portion of the kidney is excised to preserve its functionality. Analogously to RARP, the RAPN procedure can be subdivided in five main stages: (1) bowel mobilisation, (2) hilum dissection, (3) tumour identification and demarcation, (4) clamping of the hilum, and (5) tumour excision. According to surgeons, the procedure phase that benefits the most from AR is phase 3, which allows the surgeon to quickly identify endophytic and posteriorly located tumours,^42^ since during this procedure the kidney is not perfused with blood and consequently risks necrosis. Thus, the kidney was used as a target (Case Study 3).

Concerning the RARP and RAPN data at disposal, main datasets parameters, namely number of real or synthetic images for both the CNNs and rotation ranges, are resumed in Table 1. In all cases, datasets, for both segmentation and rotation, were split into 70%, 15%, 15% for train, validation and test, respectively.

**TABLE 1:** Values referring to the images used for datasets creation, both synthetic and real, for segmentation and rotation Convolutional Neural Networks

			Segmentation	Rotation	
Surgical operation	Case study	Target	# Real images	# Synthetic images	Rotation ranges in degrees (−X,+X)/(−Y,+Y)/(−Z, +Z)
RARP	1	Catheter	375	40000	(−40, 10)/‐/‐
RARP	2	Prostate	388	35000	(−15, 20)/(−25, 25)/(−5, 15)
RAPN	3	Kidney	208	40000	(−10, 10)/(−10, 10)/(−10, 10)

*Note*: For the latter, considered rotation ranges are also shown. For the catheter, the Rotation CNN was trained to predict only the X rotation, as Y was derived directly from the segmentation map and Z was considered irrelevant.

The network used for segmentation was a UNet‐ResNet architecture, trained for 50 epochs using a batch size of 4 and an Adam optimizer with a learning rate of 0.0001, while the one used for rotation was a modified version of ResNet50, trained for about 15 epochs, using a batch size of 32 and an Adam optimizer with a learning rate of 0.00001.

## 4 RESULTS

### 4.1 SegmentationCNN

**FIGURE 3:**
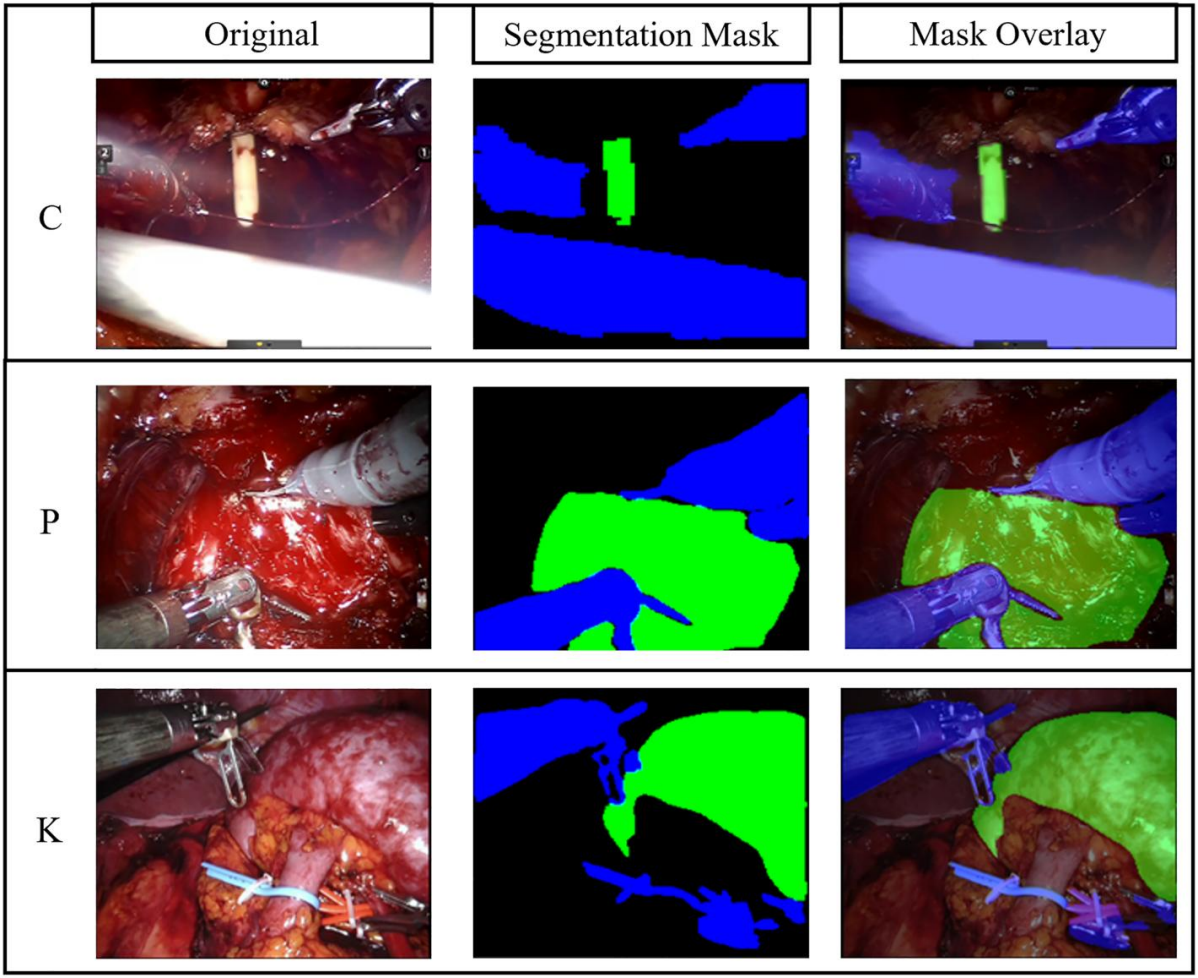
Sample images referring to original endoscopic images, segmentation masks and mask overlay (columns) for each case study (rows): catheter (C), prostate (P) and kidney (K)

The SegmentationCNN showed optimal results for all case studies, represented in Table 2. Before selecting the final architecture, as mentioned in Section 3.2, several of the most used models for semantic segmentation were compared. The final choice was made relying on the IoU metric, computed both on the individual classes (*background*, *tool*, *target*) and as an average between them. The IoU score for the target class was used as discriminating factor, since the organ identification is the main goal of the semantic segmentation task. Among all the model combinations, UNet‐ResNet was finally deemed as the best, as it showed the highest IoU score in terms of both target class (0.9450 for the catheter, 0.7296 for the prostate and 0.8602 for the kidney) and mean value (0.8940 for the catheter, 0.8067 for the prostate and 0.9069 for the kidney). In Figure 3 representative samples of the network's output masks, together with their overlay on the real images, are shown.

**TABLE 2:** IoU scores referring to the mean value of the actor classes (background, tool and target) and the target class only: catheter (C), prostate (P) and kidney (K)

	IoU score	
	Target class	Mean
C	0.9450	0.8940
P	0.7296	0.8067
K	0.8602	0.9069

### 4.2 RotationCNN

The results related to the rotations' prediction are resumed in Table 3.

**TABLE 3:** Values of mean accuracies when testing with a synthetic dataset, a real dataset and the same real dataset after fine‐tuning the network

		Accuracies [−5, +5]			Accuracies [−10, +10]			# of images
		*X*‐axis	*Y*‐axis	*Z*‐axis	*X*‐axis	*Y*‐axis	*Z*‐axis	
Synthetic dataset	C	0.9987	‐	‐	0.9987	‐	‐	3000
	P	0.9971	1	0.9983	0.9994	1	0.9994	1750
	K	0.9990	1	0.9990	0.9997	1	0.9987	3000
Real dataset	C	0.5333	0.9457*	‐	0.7333	0.9886*	‐	30
	P	0.5000	0.0000	0.2778	0.5556	0.0833	0.3611	36
	K	0.0000	0.0000	0.0000	0.0000	0.0000	0.2000	30
Real dataset (after fine tuning)	P	0.3333	0.5000	0.6667	0.7778	0.7222	0.9444	36
	K	0.0000	0.0000	0.5000	0.0000	0.0000	0.7333	30

*Note*: Each value is computed for the considered target classes: catheter (C), prostate (P) and kidney (K). The accuracies for *X*, *Y* and *Z*, were computed as the number of correct predictions over the total number of samples, using different acceptable ranges: prediction with an error in the range [−5, +5] and in the range [−10, +10].

*Y−Axis rotation values for C were retrieved from the semantic segmentation CNN.

The network was first tested with 3000 synthetic images for catheter (C), 1750 for prostate (P) and 3000 for kidney (K) generated with *Blender*. The accuracies for *X*, *Y* and *Z*, were computed as the number of correct predictions over the total number of samples, using different acceptable ranges. In the testing phase, it was decided to consider 5 degrees of tolerance and therefore a prediction was deemed wrong in case the difference between the estimate and the actual value was greater than this threshold. This was considered a good compromise as a difference of 5° does not significantly affect the visual result of the model superimposition. For the same reason, a 10° tolerance range was considered acceptable by the surgeons. Each evaluating parameter, namely, accuracies for [−5, +5] and [−10, +10] ranges, were computed for each one of the rotation axes (*X*, *Y*, *Z*) and for C, P and K classes, respectively.

This same network was then tested with 30 real images tagged manually for C, 36 for P and 30 for K.

The accuracies in this case were predictably acceptable only for the catheter. To improve the results for the prostate and the kidney, the network was subsequently fine‐tuned with 100 additional real images and tested with the same images as before.

### 4.3 Optical flow

**FIGURE 4:**
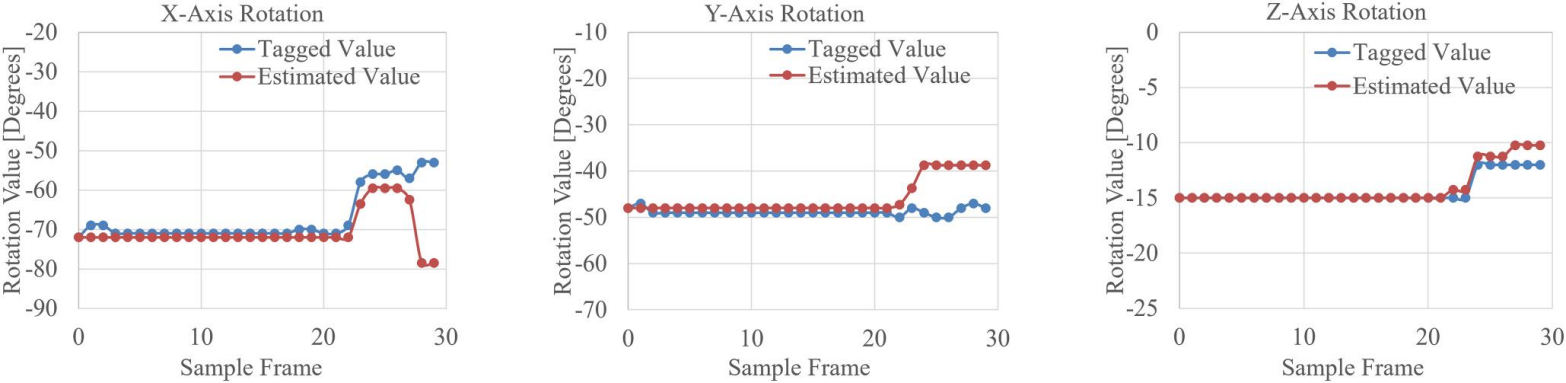
Trends representation of organ orientation on *X*, *Y* and *Z* axes comparing tagged (blue) and OF estimated (red) rotation values for each sample frame

The motion analysis algorithm was tested on an endoscopic video stream of a nephrectomy procedure fragment lasting 10 s. The results, comparing estimated and real rotation trends, are shown in Figure 4 The evaluation of the method was performed by comparing the tagged (blue values) and the inferred (red values) rotations for each axis separately. The rotation values were extracted for 30 sample frames, namely with a 10 frame‐step, adding up the rotation variations of all the in‐between frames. Analogously to the *RotationCNN* results, tolerances of 5 and 10° was considered to deem an estimation as correct.

## 5 DISCUSSION

In recent years artificial intelligence, computer vision and augmented reality technologies have spread to many fields, including surgery. In this article, the use of these technologies in supporting applications to the surgeon during laparoscopic Robot Assisted procedures was addressed. These technologies aim to augment the surgeon's perception of the surgical scene, which is usually more limited than in open procedures, as the field of view is narrowed. To mitigate this drawback, AR aims to superimpose the preoperative model on the intraoperative image, hence it is necessary to know the 6D pose of the organ.

After extensive literature analysis, both in the currently used methodologies for 6D pose estimation of an object and in the specific context of its application to AR in laparoscopic surgery, the discussed methodology was delineated. The proposed framework is a novel approach for automatic and real‐time registration of the 3D model on the endoscopic view as it relies solely on RGB data, captured by the endoscope and displayed on the screen of the robotic surgical system, with no need of further external sensors or devices. Three case studies were identified and, depending on them, different ways of handling the problem were pursued. Case Study 1 relies solely on a specific rigid instrument, the catheter. Case Study 2 and 3 dealt with soft tissue having different complexity levels.

The first phase involved a SegmentationCNN, UNet‐ResNet, for all the case studies, to distinguish the actors and obtain the target position and scale. In the second phase, a RotationCNN predicted the rotation values along the three axes, *X*, *Y* and *Z*. In the third additional phase, an OF algorithm handled the target tracking.

In Case Study 1, the approach handles both the location and rotation estimation tasks recursively for every single frame while, in Case Study 2, the rotation estimation is performed only for a starting ideal frame, then, next frames rotations are inferred through OF. These two approaches are fully automated. On the contrary, in Case Study 3, given the complexity in predicting the rotation, the registration should be manually performed before applying the OF technique.

The SegmentationCNN showed optimal accuracies, with a mean IoU score greater than 80% for all the case studies.

For the RotationCNN, accuracies for [−5, +5] and [−10, +10] ranges, were computed for each one of the rotation axes (*X*, *Y*, *Z*). Test results for synthetic images reached values close to 1, showing that the network actually learnt. To evaluate the generalisation capability of the network, real images were tested too, showing, acceptable results just in the first case study involving the catheter, aided by the fact that the Y accuracy was derived from the segmentation map. On the contrary, the results for the prostate and the kidney were not considered acceptable. Thus, fine tuning was performed with 100 additional real images for each organ. After fine‐tuning, the network underwent major improvements with the prostate, resulting in error values minor than 10° for almost every image. These tests were performed on frames where the organ was well distinguishable. On the contrary, with the kidney, the network did not improve its performances even after fine‐tuning. The only solution in this case was to perform a manual registration. Afterwards, an alternative method was included to tackle the rotation inferring task, the OF. This last approach was evaluated on an endoscopic video stream, showing that the general trend of the organ's orientation changes was correctly tracked, hence rotation events were recognized.

Nevertheless, this was the very first proposal for a generalized framework, hence there are still several limits to overcome. In particular, one of the limitations of the current application is the restricted amount of videos for the CNN training, which limits the heterogeneity of the dataset, in particular the organs have different shape and texture depending on the patient's anatomy and health condition. In addition, the case studies addressed all fall within the urological field, hence, to effectively test its generalisability, it should be broaden also to other organs such as uterus and liver. Another limitation for the generalisation of the framework refers to the heuristic that infers rotation from OF, since this was deduced from specific movement patterns of the organ. The RotationCNN performances have still room for improvement, since it still struggles in detecting the value of the rotation precisely enough to perform the tracking when dealing with soft tissues.

Considering each case study, when satisfactory results were obtained during the test phase on the endoscopic videos, in vivo tests were performed. In particular, the first Case Study, meaning when the catheter is present, was successfully tested in vivo and it worked in real‐time. For what concerns the other case studies, dealing with complex scenarios, are currently being tested, gaining positive feedback from surgeons and new insights for further improvements.

In future works, adjustments could be made in several directions. For the concerns of segmentation, instead of traditional CNNs, the novel attention technique could be exploited to focus on those pixels that are informative of the motion information, fading out the others, resulting in a faded mask rather than in a binary one. For the concerns of rotation estimation, besides testing the RotationCNN in the operating room, the heuristic inferring of the OF method should be amplified and its precision improved. Acquiring a greater number of endoscopic procedure videos could also benefit the training of the neural networks. To better exploit intraoperative information, more data should be collected, for example, an organ could have different shape and dimension depending on the patient and, the more the samples the better the ability of the neural network to improve generalisation. Furthermore, the current version of this work only used one video stream from a monocular view to reduce processing complexity, yet, in future research, a binocular vision could be used to exploit the depth information of stereoscopy. Finally, for the concerns of the real‐time tracking task, variations should be made to control whether the organ is fully framed or not, in order to correctly detect the centre of the organ under different conditions.

## 6 CONCLUSION

The current study presents a novel framework for automatic and real‐time registration of the 3D model on the endoscopic view from endoscope video flow images. The output video stream displays the endoscopic view coupled with 3D information on the screen of the robotic surgical system, with no need of further external sensors or devices.

This work exploits semantic segmentation to estimate the position, while to infer the rotation two techniques, based on Convolutional Neural Networks and motion analysis were used, depending on the surgical scenario.

The use of AR in the medical field has been widely investigated in recent years, proving that it could be an effective aid to the surgeon. Further research is required to improve the generalisation capability of this first version of the framework, and further tests are needed to consolidate real clinical advantages of AR‐aided and robot‐assisted surgery.

## CONFLICT OF INTEREST

The authors declare that they have no conflict of interest.

## Data Availability

Not applicable.
